# The Present Demand for Better Medical Education in the South

**Published:** 1892-11

**Authors:** Luther B. Grandy

**Affiliations:** Atlanta, Ga.


ATLANTA
MEDICAL AND SURGICAL JOURNAL.

VOL. IX. NOVEMBER, 1892. No. 9.

EDITED BY
LUTHER B. GRANDY, M. D., AND MILLER B. HUTCHINS, M. D.,
WITH THE CO-OPERATION OF
H. V. M. MILLER, M. D., LL. D., VIRGIL O. HARDON, M. D., AND
FLOYD W. McRAE, M. D.

### ORIGINAL COMMUNICATIONS.

### THE PRESENT DEMAND FOR BETTER MEDICAL EDUCATION IN THE SOUTH.*

By LUTHER B. GRANDY, M. D.,
ATLANTA, GA.

The twenty years that have passed have been years of unprecedented activity and progress in our art and in the sciences related thereto. It would be foreign to the scope of this paper to give even the briefest outline of the work that has been accomplished and the additional knowledge that has been gained in medicine and the collateral sciences within that period. The new world of Bacteriology has been discovered, and is just now being developed; Chemistry has received some of its most important additions; Electricity in its therapeutic relations has been studied as never before; Physiology has been revised; our Therapeutics

* Read at the meeting of the Tri-State Medical Society of Georgia, Alabama and Tennessee, Chattanooga, Tenn., October 25, 1892.

have become more rational because we have learned better of nature's processes and of the action of drugs; and the gospel of cleanliness that is next to godliness has created a new Surgery in place of the old. The French have a maxim (*plus on s'eleve, plus l'horizon s'avance*) which says that the higher we go, the broader our horizon becomes. So it is with our knowledge.

> "I doubt not through the ages one increasing purpose runs,
> And the thoughts of men are widened with the process of the suns.'›

It was not only natural but necessary that due cognizance of this progress should be taken by the institutions which had the making of a nation's doctors. Foreign schools were quick to accomplish the idea that they should improve their courses of instruction in accordance with the demands of a higher culture. In our own country the Chicago Medical College was the first to cut loose from its old moorings in inaugurating, twenty-four years ago, the graded course of instruction extending over three years. From time to time other colleges (principally those of the North and West) saw the necessity of doing likewise, and the reform movement in medical education became gradually but securely established.

The main steps in this advance movement have been:

1. The lengthening of the college term up to six months or more. In 1882 the number of colleges with this length of term was 42; in 1885 it was 50; in 1890, 76; and in 1892, about 120.

2. The requirement of preliminary education. In 1882 the number of colleges requiring certain educational qualifications for matriculation was 45; in 1886 it was 114; in 1890, 124; and in 1892, about 130.

3. The lengthening of the college course up to three years or more. In 1882 the number of colleges requiring three or more courses of lectures before graduation was 22; in 1886 it was 41; in 1890, 64; in 1892, about 100.

---

*Unless otherwise stated the statistics in this paper have either been taken from the reports of the Illinois Board of Health for 1891 and '92, or compiled from the latest college catalogues. Allowance has generally been made for prospective regulations; that is, those which will take effect only after the current session.

4. The addition to the courses of instruction of certain subjects, as hygiene, medical jurisprudence, laboratory training in normal and pathological histology, and in bacteriology, etc. Not the least important has been the requirement gradually of more and more clinical work where this was practicable.

We regret to say that the Southern colleges, as a rule, have taken too small a part in this spirit of progress. In spite of increasing demands our curricula have remained practically the same, and though the times have changed we have not changed with them.

There are in the United States and Canada to-day about 140 medical colleges of all kinds in active operation. Of these 101 belong to the regular profession in the United States. Five of these 101 colleges require four or more years of study and four or more terms of lectures as conditions of graduation; 21 require four or more years of study and three or more terms of lectures; 52 require three or more years of study and three terms of lectures; the remaining 23 either require three years of study and only two termso lectures, or only a two-term attendance upon lectures without even the trifling obligation of a previous year with a preceptor.

Three evils at present exist in the majority of our Southern schools, in the presence of which it is impossible to do good work. 1. The non-requirement of preliminary education. 2. The two-term course. 3. The laxity of our final examinations.

*I. As to Preliminary Education.* The impression once existed that if a boy betrayed unmistakable incapacity for everything else, he could at least be made a good doctor. That delusion, we believe, is now obsolete. But it is still too easy a matter to matriculate in most of our colleges. The applicant enters his name, refers possibly to his family physician as his preceptor, where this empty requirement is observed, pays his five dollars and the operation is done. All candidates are equal here, be they fresh from college halls, or fresh from field or forest. This is all wrong. The study of medicine is too difficult and responsible a task for him to undertake who is not prepared for it. The student with no knowledge of Latin, for instance, begins his course, and possibly his first lecture is on Anatomy. He hears the professor describe the *cervical*, *dorsal* and *lumbar vertebræ* with their *pedicles*, *laminæ*, *spinous* and *transverse processes*, *superior* and *inferior inter-vertebral* notches, *anterior* and *posterior tubercles*, etc., etc., and when the tedious hour is over the poor fellow cannot tell you the difference between the *vertebral foramen* and the *odontoid process*. The student with no knowledge of Latin cannot learn Anatomy and Materia Medica except with the greatest difficulty, and neither can one study Chemistry and Physiology intelligently without some previous instruction in elementary physics. It is expecting too much of students not even possessing a previous high-school training that they should be able to reach anything like an intelligent comprehension of the scientific principles involved in the practice of medicine. We cannot manufacture good doctors out of material that was designed by nature, and trained by experience, for the farm. Our attempting to do so is unjust to the "material," and only robs the farm without enriching the profession.

In England the General Medical Council now requires of matriculates a preliminary examination in the English Language, Grammar and Composition, Latin Language including Grammar and Translations from selected authors, Mathematics comprising Arithmetic, Algebra to simple equations, and Geometry through three books of Euclid, and optionally, either Greek or Logic or any Modern Language.

The Association of American Medical Colleges now demands that institutions composing it shall require of all matriculants not having a collegiate or high-school certificate of some sort "an English composition in the handwriting of the applicant, of not less than 200 words, an examination by a committee of the faculty, or other lawfully constituted Board of Examiners, in higher arithmetic, algebra, elementary physics and Latin prose." Surely this is not asking too much. This Association was organized in a Southern city more than two years ago, but how many of our Southern schools have gone into it? So far as I have been able to ascertain, only two. If there are more I shall be pleased to hear of them.

There are now about 130 medical schools in this country and Canada requiring certain educational qualifications for matriculation. Of these how many are Southern colleges? Only three. About ten colleges remain which make no effort to ascertain if the applicant is capable of receiving a medical education, and of these ten nine are in the South. In three of the best Eastern schools whose catalogues I have examined the proportion of college-bred men to the total number of matriculates averaged about 36.5 per cent. In Southern schools the corresponding proportion is only 17 per cent. It may be of some comfort for us to know that in the West the percentage is even lower than this.*

*II. As to the Two-Term Course.* The time was in the history of medical education, and within the memory of many now before me, when a few months more or less intimate association with a practicing physician, himself most likely possessing only a vague and unreliable knowledge of matters medical, and a one-term attendance upon lectures at some institution provided with the diploma-conferring power, constituted all the necessary steps in the evolution of the physician. That time is passed. It may not have been impossible in that day for a student of average capacity to master fairly well within the period required all that was offered him to learn. But the time that was sufficient to acquire a medical education twenty years ago will not suffice for the broader culture that is demanded to-day. Within a given time it is impossible for a student to accomplish more than so much. The capacity and power of medical students are limited, and the stuffing process is just as incompatible with mental, as it is with gastric, digestion.

Of the 78 American colleges which require three or more years of study and three terms of attendance upon lectures 72 are located in the North and West; the remaining 6 are in the South and three of these are schools for negroes. Of the 23 colleges which continue to graduate students after three years of study and two terms of lectures, or after two terms of lectures alone, 14 are in the five Southern States, Virginia, Georgia, Alabama, Tennessee

*DR. JOHN S. BILLINGS, in *Boston Med. and Surg. Journal*, June 25 and July 2, 1891.

and Louisiana. The list of colleges that require only two courses of lectures and have as yet made no provision for longer study is now reduced to 21. Of this number the Southern schools compose the great majority. In Italy and Switzerland the medical course covers a period of eight years ; in Denmark, seven ; in Austria, Russia and Portugal, five ; in France and Canada, four ; in the United States, four, three and two. The Association of American Medical Colleges now requires that candidates for the degree of Doctor of Medicine shall have attended three courses of graded instruction of not less than six months each in three separate years. Colleges not conforming to these minimum requirements, and the faculties of the same, will not be held in good standing by the American Medical Association. Medical Legislation is getting to be fashionable now, and every legislature that meets seems to be inspired with a patriotic desire to reform something that pertains to our profession. It would appear that these things have been hidden from the wise and prudent and revealed unto babes. The legislatures of Minnesota, Montana, North Dakota, Washington, California, Colorado, Illinois, New York, Iowa and Oregon have passed measures licensing to practice within their limits only those applicants who can certify to attendance upon three full courses of lectures of six months each—who can present a diploma of a three-term college.* There is no question that other states will sooner or later adopt similar legislation. Even in the legislature of Georgia last year there were a few feeble and flickering signs of activity in this direction over a bill requiring the medical colleges of the State to adopt the three-year course.

Without attempting to make any comparisons, we may say that possibly the four best schools in the United States are the Harvard Medical School, College of Physicians and Surgeons (New York), University of Pennsylvania, and the University of Michigan. Yet three of these now require four years of college attendance, and the other is preparing to do so. If these colleges, with their superior teaching facilities, their excellent laboratory equipments, and their abundance of dispensary and hospital ma-

*DR. GEO. J. ENGELMANN, in *Medical Fortnightly*, April 15, 1892.

terial, will not declare a student prepared to practice until he first proves his capacity to begin, and then spends four years in study and observation, what shall be said of those institutions, with their meager (relatively) opportunities, which, without question, receive all applicants that come, and after two short terms of study send them back to their constituents indorsed with the great seal as duly qualified physicians? To make the nurse in our ordinary training schools the term of instruction is usually two or more full years of constant, and oftentimes laborious, work day in and day out; to make the physician or surgeon, who is above nurse and everybody else at the bedside, requires only two terms of study of five or six months each. There is something wrong here.

Before the Examining Boards of Alabama, North Dakota, North Carolina, Virginia and Minnesota there have been 1,950 examinations for license to practice medicine. The total percentage licensed was 75.2 per cent. Of 183 graduates of colleges requiring three courses of instruction for the degree 179 were accepted; 4 were rejected; percentage passed, 97.2. Of 435 graduates of colleges that formerly issued degrees after attendance upon two courses of lectures 343 passed the examinations; 92 were rejected; percentage passed, 78.8.*

It has been well and truly said by a recent writer that those of the profession now in practice who received their degrees after attendance upon but two courses of medical instruction "now look back upon their medical college career as an unimportant epoch, and think of those days as a work of confusion. The instruction afforded by this system of medical education was hurried, superficial and most inadequate to our wants, the course consisting rather in calling the attention of the student to the art of medicine, instead of teaching him the sound principles upon which is governed the great field of active practice".†

Here is the testimony of a distinguished American physician who has been honored at home and decorated abroad for his

*DR. P. H. MILLARD, in *Medical News*, July 30, 1892.
†*Idem.*

contributions to scientific medicine and sanitation*: "Thirty-three years ago I began the study of medicine, having obtained the degree of Bachelor of Arts after the usual classical course of those days. The smattering of Latin and Greek which I had obtained was of great use to me, and I may, therefore, be a prejudiced witness but my acquaintance with many physicians at home and abroad has led me to believe that the ordinary college course in languages, mathematics, and literature is a very good foundation for the study of medicine. I had attended lectures in physics and chemistry but had done no laboratory work, and I could read easy French and German. Thus equipped I began to read anatomy, physiology and the principles of medicine. Nominally I had a preceptor, but I do not think I saw him six times during the year. Nevertheless he told me what books to read, and I read them. The next thing was to attend the prescribed two courses of lectures in a medical college Each course lasted about five months and was precisely the same. There was no laboratory course, and I began to attend clinical lectures the first day of the first course. One result of this was that I had to learn chemical manipulation, the practical use of the microscope, etc., at a later period when it was much more difficult. In fact I may say that I have been studying ever since to repair the deficiencies in my medical training and have never been able to catch up." It may now be pertinently asked, wherein does the course which he pursued thirty-three years ago differ from that now followed by students in the majority of our Southern Colleges?

*III. As to Final Examinations.* The requirements at the close of our sessions would seem to be hardly more difficult than those at the beginning. In consideration of the fact that many states now have Examining Boards before whom the graduates of the colleges must appear, would it not be to the interest of the colleges to send out only such men as are well qualified? They would at least be spared the humiliation of seeing large numbers of their graduates rejected. Again, in consideration of the fact that many states as yet have no Examining Boards but accept a

*DR. JOHN S. BILLINGS, *Op. cit.*

diploma as a license, what becomes of the duty which a college sustains to the people when it sends into an unprotected community one or more men totally unfit to practice? It is easy to conceive when a little learning may be a dangerous thing in medicine or surgery. A graduate of one of our Southern colleges that stands as well with us, I am sorry to say, as any other made a record before the Virginia Examining Board last April of 85 per cent. on Surgery, 78 on Materia Medica, 65 on Practice, 50 on Physiology, 50 on Chemistry, 35 on Hygiene and Medical Jurisprudence, 34 on Anatomy, and 33 on Obstetrics. Before the same board graduates of other colleges, North and South, made some shamefully low averages. One man made only 54 in Chemistry, 53 in Physiology, and 52 in Anatomy; another, 38 in Anatomy and 30 in Physiology; another 25 in Anatomy; another 22.* And yet, these men all had medical college diplomas. In many of the colleges from which these graduates come, their deficiencies in one or two branches are allowed to be compensated for by unexpected excellencies in others. Thus a student who attains only 50 per cent. in Surgery and Obstetrics may be passed on account of his good marks in Chemistry and Materia Medica; but, we may ask, does his superior record in the latter attest his ability to reduce a dislocation or manage a natural or unnatural labor? One trouble is that professors in the regular faculties do not know their students well enough to learn their deficiencies. It is the assistant intructors who make the demonstrations in the different departments, conduct the college quizzes, and are in daily touch with the student body that are in position to know that many of them do not earn their diplomas; in fact, have no right to them. But these same assistants have no voice in the final acceptance or rejection of candidates for graduation. The man who in two years time has not learned better than to say that the foramen magnum transmits the arch of the aorta has no right to a diploma. But such a man is now at large somewhere rejoicing in the possession of a degree bestowed upon him by one of our colleges.

Every department of a medical course should presuppose, as a

*Report of Virginia Examining Board for first session of 1892.

condition requisite, a reasonable amount of education in the English essentials, and yet we are yearly making doctors of those who cannot write a correct English sentence. Those in this audience who conduct college correspondence will bear me out in that statement. Any medical course that makes any pretensions to thoroughness is now comprehensive enough to make it impossible for a student of only average attainments to obtain a safe working education in two terms of six months each, and yet two thirds of our Southern schools require no more; some do not require that much. What sort of doctors are we to expect?

The Association of American Medical Colleges, after a somewhat checkered career, held its third annual session in Chicago last summer, and two-thirds of the medical schools of the country were represented. The South was not there. This incident excited the following comment in a popular Northern journal (*Medical Record*):

"We trust that our brethren in the South will appreciate the situation: Their colleges are confessedly poor, and there can be no advance because the cheaper ones outbid the others. Southern physicians are sensitive to the imputation of commercialism, and they are justly proud of their capacity and resources as educators. We trust that they will speedily take means to correct the opinion which must now be frankly expressed, that Southern medical colleges are cheap colleges, not necessarily because they are inferior in equipment, but because they are low in their requirements."

This is a condition, not a theory. I have presented in this paper a rugged array of facts, and have added no gaudy embellishments of fancy.

There was a time when medicine was the most learned profession next to the priesthood. The title *Doctor* was then bestowed not upon those who practiced medicine and surgery, but was reserved for that limited few to whom it was permitted to enter the Holy of Holies of classical and scientific learning. By extension the term came to be applied also to the followers of the healing art, the exigencies of whose calling demanded a degree of knowledge second only to that of the monks in the monasteries. With the profession and with the colleges rests the keeping of our ancient prestige. Let us see that none of it is lost.

We, as medical men, sometimes take a pride in saying that it is not all of practice to make money; so let it be said of our colleges. Instead of that mercantile spirit of competition which seeks to catalogue the greatest number of students there should be an unselfish spirit of rivalry in giving those students that are honorably attracted the most useful and thorough preparation for their lifework. The college that best succeeds in doing this will not want for patronage; but will quickly achieve the happy distinction of giving the greatest good to the greatest number.

